# The efficacy of cariprazine in chronic schizophrenia – post hoc analyses of phase II/III clinical trials

**DOI:** 10.1192/j.eurpsy.2022.822

**Published:** 2022-09-01

**Authors:** P. Falkai, Z. Dombi, K. Acsai, Á. Barabássy, G. Németh

**Affiliations:** 1 Ludwig-Maximilians-Univeritiät Munich, Department Of Psychiatry And Psychotherapy, Munich, Germany; 2 Gedeon Richter Plc, Medical Division, Budapest, Hungary

**Keywords:** cariprazine, schizophrénia

## Abstract

**Introduction:**

Chronic schizophrenia patients are experiencing persistent and severe illness for more than 15-20 years and are usually suffering from long-term negative symptoms. Cariprazine, a novel D_3_-D_2_ partial agonist has been proven to be effective in the treatment of acute schizophrenia, however its ability to treat chronic patients has not been assessed yet.

**Objectives:**

The primary aim of the present post-hoc analysis is to assess the efficacy of cariprazine in treating patients with chronic schizophrenia (late-stage and residual schizophrenia patients).

**Methods:**

Data from 3 phase II/III 6-week, randomized, double-blind, placebo-controlled trials with similar design in patients with acute exacerbation of schizophrenia were pooled and patients with more than 15 years of schizophrenia were analysed (late-stage patients). Furthermore, schizophrenia patients experiencing predominantly negative symptoms from a 26-week, randomized, double-blind, active-controlled, fixed-flexible-dose trial with an ICD-10 code of F20.5 were analysed post-hoc (residual patients).

**Results:**

Altogether, 414 late stage (286 cariprazine and 128 placebo) and 35 residual (23 cariprazine and 12 risperidone) patients were identified. The pooled analysis evaluating mean change from baseline to week 6 in the PANSS total score indicated statistically significant difference in favour of cariprazine in the late stage (LSMD -6.7, p<0.01) subpopulation compared to placebo. The mean change from baseline in patients with residual schizophrenia in the cariprazine arm was -9.6 on the PANSS-FSNS scale, while -7.9 in the risperidone arm.

**Conclusions:**

Based on the results, it seems that cariprazine might be a good treatment option for patients with chronic schizophrenia. Nonetheless, further studies are needed to confirm this.

**Disclosure:**

I am an employee of Gedeon Richter Plc.

